# Retracted: The Effects of Fetal Gender on Serum Human Chorionic Gonadotropin and Testosterone in Normotensive and Preeclamptic Pregnancies

**DOI:** 10.1155/2013/518591

**Published:** 2013-11-02

**Authors:** Journal of Pregnancy

The paper titled “The Effects of Fetal Gender on Serum Human Chorionic Gonadotropin and Testosterone in Normotensive and Preeclamptic Pregnancies” [1], published in Journal of Pregnancy, has been retracted as it is found to contain a substantial amount of material from the paper “Human chorionic gonadotropin and testosterone in normal and preeclamptic pregnancies in relation to fetal sex,” authored by Johan Arnt Steier, Magnar Ulstein, and Ole L. Myking, and it is published in “Obstetrics & Gynecology” in September 2002.
